# Three-dimensional distensibility of the aorta derived from four-dimensional cardiovascular magnetic resonance in young and middle-aged adults with Marfan syndrome^☆^

**DOI:** 10.1016/j.jocmr.2025.101975

**Published:** 2025-10-29

**Authors:** Daan Bosshardt, Renske Merton, Bibi A. Schreurs, Roland R.J. van Kimmenade, Aart J. Nederveen, Moniek G.P.J. Cox, Arthur J.H.A. Scholte, Eric M. Schrauben, Gustav J. Strijkers, Vivian de Waard, Daniëlle Robbers-Visser, Maarten Groenink, Pim van Ooij

**Affiliations:** a Radiology and Nuclear Medicine, Amsterdam University Medical Center, Amsterdam, the Netherlands; b Amsterdam Cardiovascular Sciences, Amsterdam, the Netherlands; c Cardiology, Amsterdam University Medical Center, Amsterdam, the Netherlands; d Cardiology, St. Radboud University Hospital, Nijmegen, the Netherlands; e Cardiology, University Medical Center Groningen, Groningen, the Netherlands; f Cardiology, Leiden University Medical Center, Leiden, the Netherlands; g Biomedical Engineering and Physics, Amsterdam University Medical Center, Amsterdam, the Netherlands; h Medical Biochemistry, Amsterdam University Medical Center, Amsterdam, the Netherlands

**Keywords:** Marfan, 4D CMR, Aorta, 3D distensibility, Displacement

## Abstract

**Background:**

Acute aortic syndromes in Marfan syndrome (MFS) often occur before reaching the surgical diameter threshold, highlighting the need for new imaging biomarkers.

**Objectives:**

Aim was to compare cardiovascular magnetic resonance (CMR)-derived aortic three-dimensional (3D) distensibility and displacement in MFS patients with or without a history of aortic root surgery (RR or native) and healthy volunteers.

**Methods:**

The participants underwent 3T CMR of the thoracic aorta using an accelerated non-contrast-enhanced, free breathing, 3D cine balanced steady state free precession sequence, with spatiotemporal resolution: (1.0 mm)^3^/∼33 ms. A deep learning-based algorithm was used to obtain aorta segmentations. Non-rigid registration of these segmentations was subsequently used to calculate 3D distensibility and its separate components: 2-dimensional distensibility, longitudinal strain, and displacement in the ascending (AAo) and descending aorta (DAo).

**Results:**

Forty-seven volunteers, 51 native, and 33 RR MFS patients were included. AAo and DAo distensibility (10^-3^*mmHg^-1^) were different for healthy volunteers vs native vs RR patients (AAo: 5.1±1.4 vs 3.6±1.4 vs. 1.4±0.7, *p<0.001*, DAo: 3.2±1.1 vs. 2.5±0.9 vs 2.4±1.0, *p = 0.001)*. Sinotubular junction displacement (mm) was significantly higher for healthy volunteers vs native MFS vs RR MFS patients (10.3±1.3 vs 8.7±2.1 vs 5.7±1.6, *p<0.001*). In native patients, age (β *= −0.06 (95% CI:−0.10 to −0.01), p= 0.014*) and root diameter (β *= *-0.1 (95% CI: −0.19 to −0.02), *p = 0.018*) were negatively associated with AAo 3D distensibility, independent of male sex, body surface area, and aortic tortuosity index.

**Conclusion:**

Aortic 3D distensibility and displacement, derived from 4-dimensional CMR, were significantly diminished in MFS compared to volunteers and should be investigated longitudinally to assess their potential value in predicting aortic events and guiding therapy.

## Graphical abstract

**Figure:**
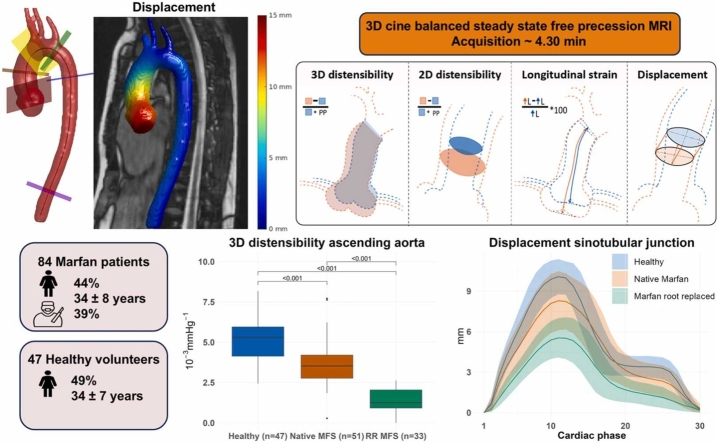

## 1. Introduction

Marfan syndrome (MFS) is an autosomal dominant, inheritable connective tissue disorder affecting 1 in 5 to 10,000 individuals [1]. Aortic aneurysm formation with a risk of aortic dissection is a major manifestation of MFS and is the main reason for increased mortality in the MFS population [2]. Aortic diameter is currently the only criterion to guide aortic surgery in MFS [3]. Although aortic diameter is a strong predictor for aortic dissection in the ascending aorta (AAo), type A dissections before reaching the surgery threshold still occur [4]. More importantly, the value of aortic diameter as a predictor for dissections in the descending aorta (DAo), is poor [5]. Therefore, new imaging biomarkers are required to guide therapy and prevent aortic events.

In MFS, a mutation in the FBN1 gene alters the extracellular matrix integrity of the aortic wall through tunica media and adventitia degeneration, which results in elevated arterial stiffness and plays a major role in the development of aortic dissection [6], [7], [8]. Distensibility, a measure of arterial stiffness, describes the relative change in aortic dimensions with pressure changes, usually assessed in static two-dimensional (2D) planes [9]. However, as the heart contracts and blood is ejected, the aorta undergoes cyclic motion, leading to displacement and deformation in the AAo and DAo. Measuring distensibility in a static plane might therefore not reflect the actual deformation. Capturing this requires three-dimensional (3D) visualization with high spatiotemporal resolution, and allows for the evaluation of 3D distensibility, which incorporates aortic dilatation, displacement, and elongation in one single parameter.

We have recently developed a non-contrast enhanced 3D cine balanced steady state precession free (bSSFP) cardiovascular magnetic resonance imaging (CMR) sequence and a deep learning-based automatic segmentation workflow, which can be used to visualize and quantify 3D displacement in the thoracic aorta with good repeatability and reproducibility [10], [11]. The aim of this work is to evaluate 3D distensibility in MFS patients in comparison with healthy volunteers (HV) and relate this parameter to current known risk factors for disease severity. Additionally, we aimed to investigate the separate components of 3D distensibility: 2D distensibility, longitudinal strain, and displacement.

## 2. Methods

### 2.1. Study design and participants

The current study had a cross-sectional design. Patients and HV were prospectively enrolled from February 2023 to June 2024. Patients were identified in four Dutch hospitals with a specialized multidisciplinary Marfan screening clinic. Eligible patients were aged 18–50, diagnosed with MFS according to the revised Ghent criteria, with a known FBN1 mutation, without a contra-indication for the study MRI, without aortic surgery beyond the AAo, an internal cardiac defibrillator, or pacemaker. [12]. Age- and sex-matched HV without a history of cardiovascular disease were recruited via social media and a local recruitment platform. Patients over 50 were not eligible in to limit the influence of age-related cardiovascular disease on the observations. All study examinations were performed at one center (Amsterdam UMC). The local ethics boards approved the study, and written informed consent was obtained from all participants. Three groups were defined as follows: HV, MFS patients without and with a history of aortic root surgery (native and RR MFS).

### 2.2. Data acquisition

**Fig. 1:**
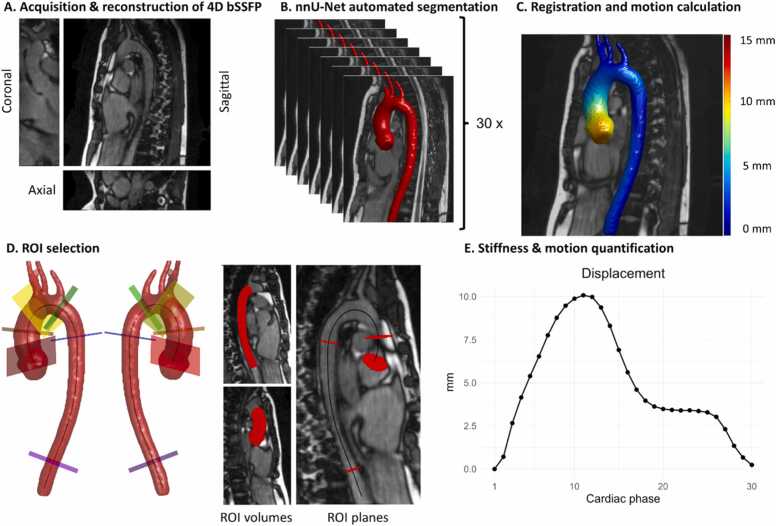
Acquisition and postprocessing pipeline. A) Acquisition of a 3D cine bSSFP scan with compressed sensing reconstruction into a 4-dimensional dataset. B) Automated segmentation of 30 cardiac phases using a trained nnU-net model. C) Registration of an end-diastolic reference phase to the other 29 phases and calculation of displacement. D) Selection of six perpendicular planes along the centerline to define four aorta planes and two volumes. E) Quantification of metrics of interest in these planes and volumes. *3D* three-dimensional, *bSSFP* balanced steady state free precession, *ROI* region of interest

An overview of the CMR acquisition and postprocessing pipeline is presented in Fig. 1. All participants underwent imaging of the thoracic aorta using a non-contrast-enhanced, free breathing 3D cine bSSFP sequence on an Ingenia 3.0T magnetic resonance imaging scanner (Philips Healthcare, Best, Netherlands) [10]. PROspective Undersampling in multiple Dimensions acceleration was used with a field-of-view (FOV) size tailored to the patient size to cover the anterior-posterior chest wall dimension and right-left extent of the thoracic aorta [13]. The median (IQR) scan time was 4:27 min (4:21–4:55) and depended on the FOV and arrhythmia rejection, resulting in an acceleration factor of R_proud_ ∼ 19 [13]. Electrocardiography or photoplethysmography was used for retrospective cardiac binning. To correct respiratory motion, automated self-gating was used to retrieve the respiratory signal and retrospectively register and average four respiratory bins to expiration [14]. Scan parameters were FOV = 256 × 256–320 × 70–88 mm^3^, slice oversampling factor = 1.70–2.14; acquired/reconstructed spatial resolution = (1.6/1.0 mm)^3^ isotropic, reconstructed to 30 cardiac phases, repetition time = 2.90 ms, echo time = 1.44 ms, flip angle = 40°, and a sinc-Gauss radiofrequency pulse shape with one sinc-period.

Additionally, a clinically used mid-diastolic 3D mDixon scan with isotropic spatial resolution of 0.75 mm was acquired to measure the aortic diameter in planes manually placed on four predefined levels: the aortic root (at the level of the sinus of Valsalva, maximum of cusp-to-cusp distance); the AAo and proximal DAo at the level of the pulmonary artery bifurcation, and the distal DAo at the level of the diaphragm.

Data on baseline characteristics and medication use were retrieved from the electronic patient records and a questionnaire.

Peripheral blood pressure and heart rate were recorded non-invasively from the brachial artery using a Microlife BP A100 Plus (Microlife AG, Widnau, Switzerland) oscillometric sphygmomanometer. The mean of two measurements before and one after the CMR examination was used for analysis.

Reports from the most recent clinically acquired transthoracic echocardiograms were assessed to determine the presence of left ventricular dysfunction and aortic regurgitation in the two patient groups.

### 2.3. Postprocessing

A previously published deep learning nnU-net automatic segmentation network was used to segment the aorta in all cardiac phases [11], [15]. For the current analysis, the network was trained on 87 manual aorta segmentations of varying cardiac phases of 74 randomly selected participants enrolled in the study (13 volunteers and 61 MFS patients). All scans and automatically segmented aortas were manually evaluated and optimized or excluded in the case of insufficient data quality, if necessary.

The time-resolved segmentations were used to derive four-dimensional (4D) aortic diameter maps. Displacement maps were created using registration of a single reference end-diastolic segmentation to all other cardiac phases segmentations and calculating the Euclidean distance traveled per segmentation surface point (Fig. 1C) [10], [16], [17]. End-diastole was defined as the first cardiac phase of the cardiac cycle, as reported by the MR compatible electrocardiography or by visually assessing the last phase without ventricular contraction for photoplethysmography datasets. After extraction of the centerline, an extension to a manually selected starting point at the level of the annulus was annotated, and after smoothing of the centerline through the aorta, four planes were manually placed on predefined levels (Fig. 1D): 1) The sinotubular junction, or equivalent level for RR MFS patients; 2) the AAo at the level of the pulmonary artery bifurcation; 3) the proximal DAo at the level of the pulmonary artery bifurcation and 4) the distal DAo at the level of the diaphragm. Two additional planes were indicated to define the AAo, from the aortic annulus to the brachiocephalic trunk, and the DAo from the left subclavian artery to the level of the diaphragm (Fig. 1D).

**Fig. 2:**
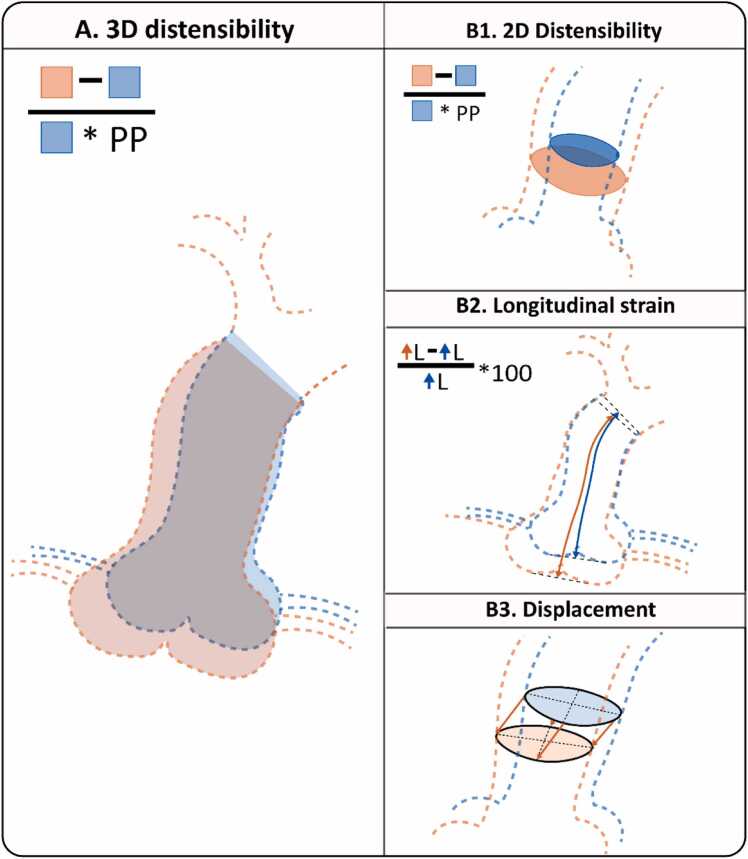
Overview of CMR parameters. All parameters were acquired comparing the phase with the highest difference from end-diastole (cardiac phase 1): A) 3D distensibility of the AAo and DAo B1) 2D Distensibility for the mid-ascending and descending plane corrected for through plane motion B2) Longitudinal strain of the AAo B3) 3D displacement magnitude of four planes in the thoracic aorta. *CMR* cardiovascular magnetic resonance, *3D* three-dimensional, *AAo* ascending aorta, *DAo* descending aorta, *2D* two-dimensional

Fig. 2 summarizes all acquired parameters. 3D distensibility was defined as $\frac{V_{\max}-V_{\mathrm{end}-\mathrm{diastole}}}{V_{\mathrm{end}-\mathrm{diastole}}*\mathrm{PP}}$ with V_max_, the maximum volume over the cardiac phases, V_end-diastole_ volume at end-diastole and *PP* peripheral pulse pressure (Fig. 2A). This was calculated for the AAo and DAo volume and shown next to dynamic volumetric change graphs over the cardiac cycle. 2D distensibility was calculated for the mid-ascending and mid-descending planes, taking into account their motion, and defined as $\frac{A_{\max}-A_{\mathrm{end}-\mathrm{diastole}}}{A_{\mathrm{end}-\mathrm{diastole}}\cdot \mathrm{PP}}$, with A_max_ the maximum aortic area over the cardiac phases, A_end-diastole_ the area at end-diastole, and peripheral pulse pressure (Fig. 2B1). The areas were calculated by finding the intersection of the perpendicular plane with the segmentation mask and then calculating the area. Longitudinal strain was defined for the AAo volume as the centerline length change over the cardiac cycle $\frac{L_{\mathrm{card}}-L_{\mathrm{end}-\mathrm{diastole}}}{L_{\mathrm{end}-\mathrm{diastole}}}*100\%$, with L_card_ the AAo length from annulus to the brachiocephalic trunk for the corresponding cardiac phase and L_end-diastole_ the length at the first cardiac phase (Fig. 2B2). Absolute mean displacement was calculated for each of the planes (Fig. 2B3).

For all parameters, the maximum values or changes compared to end-diastole (cardiac phase 1) of each subject were used for comparative analysis between groups.

Aortic tortuosity was evaluated using the aortic tortuosity index (ATI), and was automatically calculated by dividing the end-diastolic centerline length from the sinotubular junction to the diaphragm plane by the geometric distance between the two planes [18].

### 2.4. Statistics

A one-way ANOVA was used to compare differences in continuous baseline and CMR parameters between native MFS, RR MFS, and HV. Bonferroni correction for multiple testing was used for the CMR parameter analyses. Analysis of variance (ANOVA) p-values <0.005 were considered statistically significant. For significant CMR parameters, pairwise comparisons were done using Tukey-honestly significant difference (HSD) tests. Categorical values were compared using an X^2^ test. Linear regression was conducted as an exploratory analysis to examine relevant baseline characteristics (Medication use, sex, left ventricular function, aortic regurgitation, FBN1 variant type, age, blood pressure, mean arterial pressure, body surface area, heart rate, aortic root diameter, aortic tortuosity index, years since root surgery and type of root replacement procedure) in relation to 3D distensibility. Parameters with a *p*-value <0.05 in the univariate analysis were entered in multivariate models. A variance inflation factor >5 was used as a threshold for the detection of multicollinearity [19]. Pearson correlation coefficients (r) were used to assess the relationships between 2D and 3D distensibility measurements. For comparison of baseline characteristics, Tukey-HSD tests, and exploratory analyses, a *p*-value <0.05 was considered statistically significant.

## 3. Results

### 3.1. Study participants

**Fig. 3:**
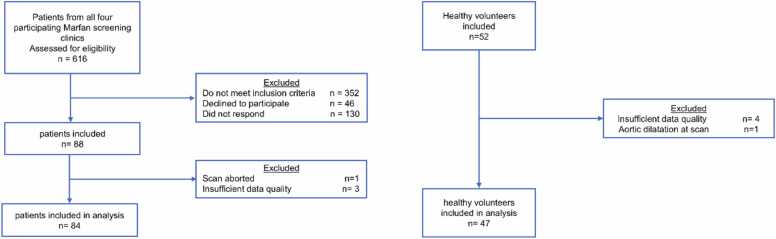
Inclusion flowcharts

The inclusion flowcharts are presented in Fig. 3. Of the 616 screened MFS patients, 272 fulfilled the inclusion criteria. 88 MFS patients and 52 healthy volunteers signed informed consent and underwent the study CMR. Seven participants were excluded due to insufficient scan quality (four due to banding artifacts in the aorta, three due to insufficient signal in the blood pool of the aorta). One patient was excluded after the CMR examination could not be completed due to claustrophobia. One RR MFS patient had atrial fibrillation during the study CMR; however, scan quality was sufficient for data analysis. Previously undiagnosed aortic dilatation was found during the CMR examination in one HV, who was therefore excluded. Characteristics of the remaining 84 patients (39% (33/84) with a history of aortic root surgery) and 47 HVs are summarized in Table 1. Height, weight, body surface area (BSA), heart rate, aortic diameters, and ATI were significantly different between the studied groups. Available echocardiography was acquired at a median of 321 (IQR: 490 - 150) before the CMR study. Presence of aortic regurgitation was more common in the RR MFS vs native MFS group (24% (8/33) vs 10% (5/51), *p*=0.031).”

**Table 1:** Participant characteristics.

	Healthy volunteers (n = 47)	Native MFS patients (n = 51)	RR MFS patients (n = 33)	*p-value Group differences*
Age at inclusion (y) [range]	34 [22–50]	34 [19–50]	36 [24–50]	*0.474*
Sex (female)	23 (49%)	26 (51%)	11 (33%)	*0.246*
Height (cm)	178±11	188±10	193±10	***<0.001***
Weight (kg)	75±12	80±14	86±17	***0.002***
BSA (m^2^)	1.9±0.2	2.0±0.2	2.1±0.2	***<0.001***
BMI (kg/m^2^)	24±3	23±3	23±4	*0.344*
Systolic BP (mmHg)	128±13	128±11	127±10	*0.905*
Diastolic BP (mmHg)	81±9	81±7	79±7	*0.498*
Mean arterial pressure (mmHg)	97±9	96±8	95±8	*0.662*
Pulse pressure (mmHg)	48±8	47±7	48±6	*0.847*
Heart rate (beats/min)	65±9	71±12	67±9	***0.016***
Betablocker use	-	19 (37%)	20 (61%)	*0.061*
ARB use	-	29 (57%)	19 (58%)	*1.000*
Betablocker & ARB	-	12 (23%)	11 (33%)	*0.463*
FBN1 Mutation effect				*0.099*
Haploinsufficient	-	12 (24%)	15 (46%)	
Dominant negative	-	28 (55%)	14 (42%)	
Effect unknown	-	11 (22%)	4 (12%)	
Bicuspid aortic valve	0 (0%)	3 (6%)	0 (0%)	-
Left ventricular function (TTE)				*0.073*
Normal	-	49 (96%)	27 (82%)	
Mildly decreased	-	2 (4%)	6 (18%)	
Aortic insufficiency	-			***0.031***
None or trace	-	46 (90%)	25 (76%)	
Mild	-	3 (6%)	8 (24%)	
Moderate	-	2 (4%)	0 (0%)	
Root surgery procedure				
Valve-sparing	-	-	17 (52%)	
Bentall	-	-	13 (39%)	
PEARS	-	-	3 (9%)	
Aortic diameters				
Root (mm)	33±4	43±5	**-**	***<0.001***
Ascending (mm)	30±4	31±4	**-**	*0.438*
Proximal descending (mm)	23±3	26±4	29±4	***<0.001***
Distal descending (mm)	20±2	21±2	24±4	***<0.001***
Aortic diameters BSA indexed				
Root (mm/m^2^)	17±2	21±2	**-**	***<0.001***
Ascending (mm/m^2^)	16±2	15±2	**-**	***0.048***
Proximal descending (mm/m^2^)	14±1	13±1	14±3	***0.002***
Distal descending (mm/ m^2^)	12±1	10±1	14±2	***0.001***
Aortic tortuosity index	2.3±0.2	2.5±0.2	2.5±0.3	***0.045***

Variables presented as (mean +/- SD) or mean [range] or n (%). *ARB* angiotensin-II receptor blocker, *BMI* body mass index, *BP* blood pressure, *BSA* body surface area, *Native MFS* Marfan syndrome patient without a history of aortic root surgery, *PEARS* personal external aortic root support, *RR MFS* Marfan syndrome patient with a history of aortic root surgery

### 3.2. CMR analysis

**Fig. 4:**
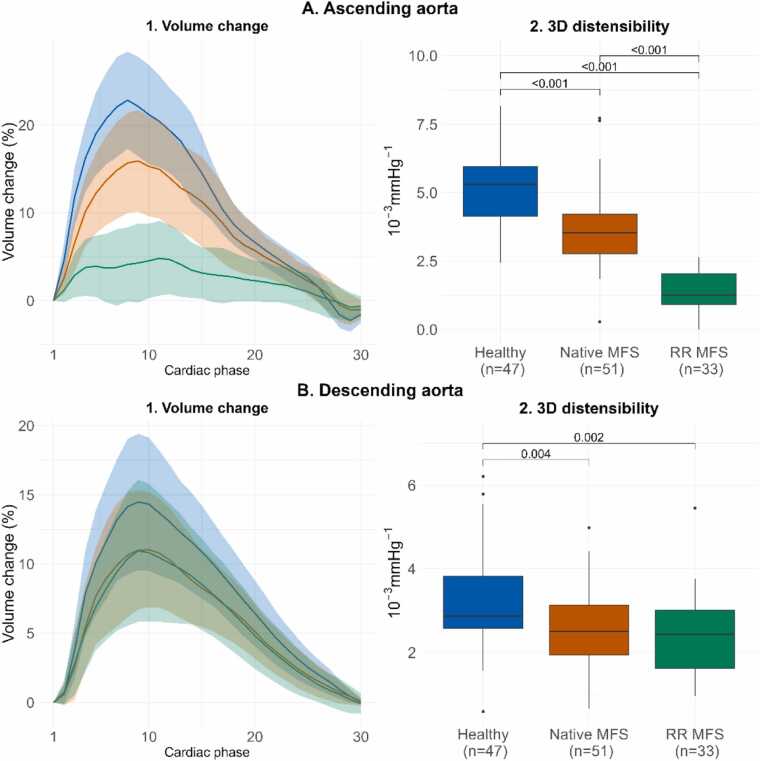
Volume change through the cardiac cycle and 3D distensibility 3D aortic volume change in the A) ascending aorta B) descending thoracic aorta, through the cardiac cycle expressed as mean +/- SD per group (A1, B1) and boxplots with median 3D distensibility with interquartile range (A2, B2). *3D* three-dimensional

**Fig. 5:**
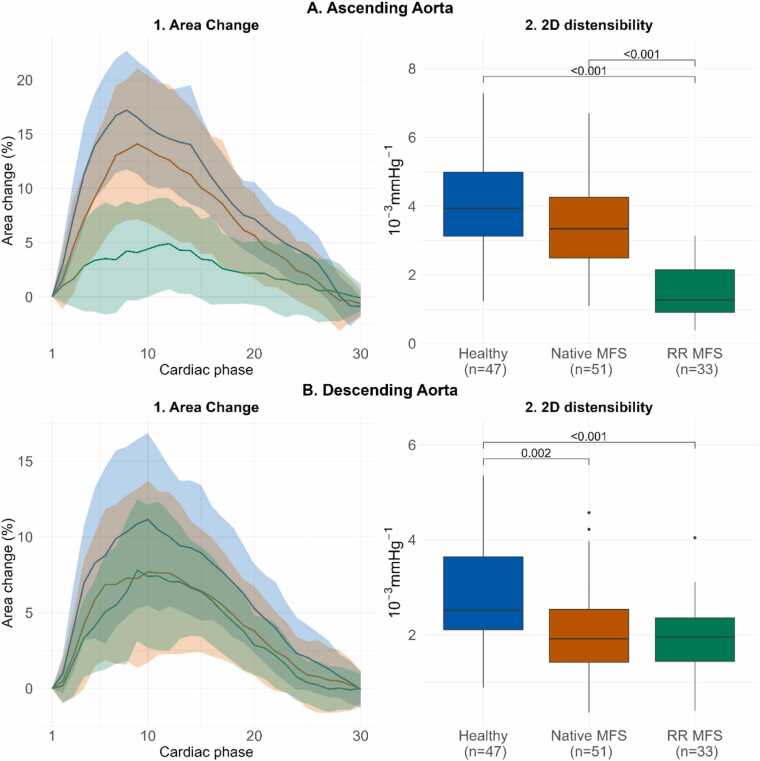
Area change through the cardiac cycle and 2D distensibility. 2D aortic area acquired in the A) mid-ascending aorta B) and mid-descending aorta at the level of the pulmonary artery bifurcation, through the cardiac cycle expressed as mean +/- SD per group (A1, B1) and boxplots with median 2D distensibility with interquartile range (A2, B2). *2D* two-dimensional, *SD* standard deviation

**Fig. 6:**
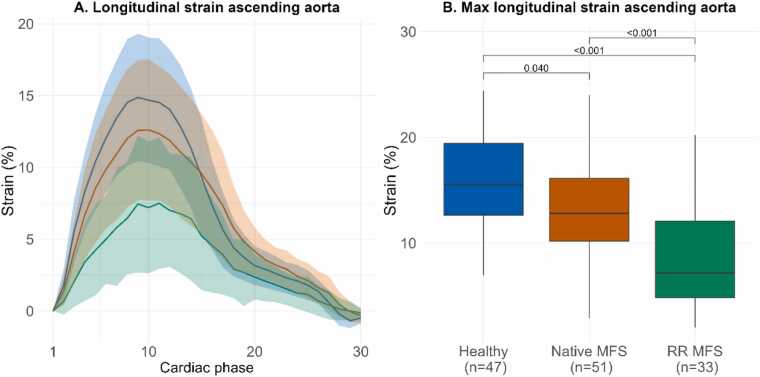
Longitudinal strain. Longitudinal strain of the in the ascending aorta per group A) through the cardiac cycle expressed as mean +/- SD and B) boxplots with median max values with interquartile range. *SD* standard deviation

An overview of all acquired parameters is presented in Table 2, and Fig. 4, Fig. 5, Fig. 6 display the different CMR parameters through the cardiac cycle and peak values. AAo 3D distensibility was significantly lower for RR MFS vs native MFS patients (*p<0.001*) and HV (*p<0.001*), and significantly lower for native MFS patients vs HV (*p<0.001*). DAo 3D distensibility change was lower for native MFS (*p = 0.004*) and RR MFS (*p = 0.002*) vs HV, but no differences were observed between the two MFS categories (*p = 0.798*).

**Table 2:** CMR parameters per group.

	Healthy (n = 47)	Native MFS (n = 51)	RR MFS (n = 33)	
	Mean±SD	Mean±SD	Mean±SD	Anova *p*-value
3D distensibility				
Ascending aorta (10^−3^*mmHg^−1^)	5.1±1.4	3.6±1.4	1.4±0.7	***<0.001***
Descending aorta (10^−3^*mmHg^−1^)	3.2±1.1	2.5±0.9	2.4±1	***0.001***
End-diastolic aortic volume				
Ascending aorta (mL)	54.3±17.3	77±23.6	73.6±15.5	***<0.001***
Descending aorta (mL)	60.4±16.6	69.9±18.1	89.9±21.1	***<0.001***
Volume change				
Ascending aorta (mL)	12.3±3.2	12.2±4.1	4.8±2.4	***<0.001***
Descending aorta (mL)	8.7±2.8	8.0±3.2	9.7±3.1	*0.046*
2D distensibility				
Mid-ascending (10^−3^*mmHg^−1^)	4.2±1.5	3.5±1.5	1.5±0.8	***<0.001***
Proximal Descending (10^−3^*mmHg^−1^)	2.9±1.2	2.1±1.0	1.9±0.8	***<0.001***
Longitudinal strain ascending aorta				
End-diastolic centerline length (mm)	79.5±9.7	90.7±11.3	83.5±13.3	***<0.001***
Centerline length change (mm)	12.4±3.4	12.0±4.5	7.3±4.3	***<0.001***
Longitudinal strain (%)	15.7±4.4	13.4±4.8	8.7±4.8	***<0.001***
Displacement (mm)				
Sinotubular junction (mm)	10.3±1.3	8.7±2.1	5.7±1.6	***<0.001***
Mid-ascending (mm)	5.1±1.1	5.1±1.3	4.5±1.3	*0.055*
Proximal descending (mm)	1.4±0.4	1.3±0.4	1.3±0.5	*0.299*
Diaphragm (mm)	2.1±0.7	2.1±0.7	1.6±0.7	*0.006*

Parameters expressed as mean +/- SD. *Native MFS* Marfan syndrome patient without a history of aortic root surgery, *RR MFS* Marfan syndrome patient with a history of aortic root surgery, *3D* three-dimensional, *2D* two-dimensional

AAo 2D distensibility was significantly lower for RR MFS vs native MFS patients (*p<0.001*) and HV (*p<0.001*). A trend towards decreased AAo 2D distensibility was observed for native MFS patients vs HV (*p<0.063*). In the DAo, 2D distensibility was different for native MFS (*p = 0.002*) and RR MFS (*p<0.001*) compared to HV, but not between the MFS categories (*p = 0.746*).

AAo longitudinal strain was significantly lower for RR MFS vs native MFS patients (*p<0.001*) and HV (*p<0.001*), and significantly lower for native MFS patients vs HV (*p = 0.040*).

**Fig. 7:**
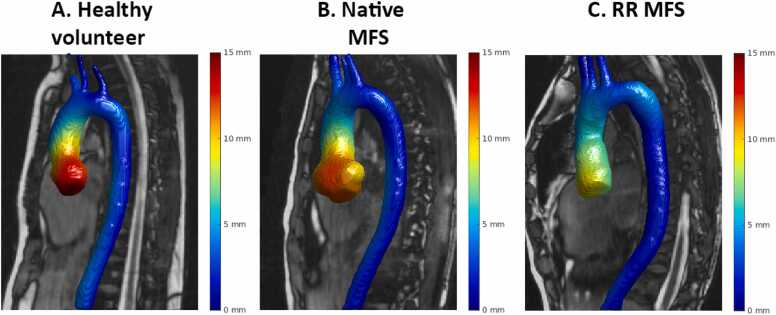
Displacement heatmaps. Maximum displacement heatmaps, compared to end-diastole for A) a healthy volunteer, B) a MFS patient with a native aortic root, and C) A MFS patient with a history of RR. *MFS* Marfan syndrome, *RR* aortic root surgery

**Fig. 8:**
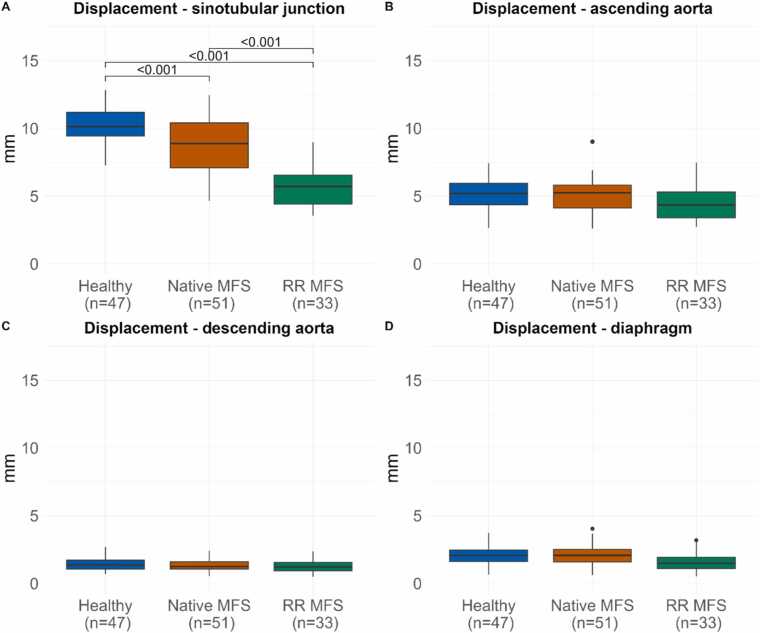
DisplacementBoxplots of median and interquartile range of maximum displacement at the level of the A) sinotubular junction B) mid-ascending, C) mid-descending, and D) diaphragm.

Fig. 7 shows a representative image of peak aortic displacement for each group (GIF available in supplementary material 1). Displacement at the four evaluated aortic levels is presented in Fig. 8. Sinotubular junction displacement was significantly lower for native MFS (*p<0.001*) and for RR MFS patients (*p<0.001*) compared to HV, but was not different between groups on the other three levels.

### 3.3. Regression analysis 3D distensibility

All linear regression analyses of baseline parameters in relation to AAo and DAo 3D distensibility are available in supplementary Tables 1–3. In the multivariate analysis, age (β = −0.06 (95% CI: −0.10 to −0.01), *p = 0.014*) and aortic root diameter (β = −0.1 (95% CI: −0.19 to −0.02), *p = 0.018*) were independently negatively associated with maximum AAo aortic 3D distensibility, while male sex (β = −0.48 (95% CI: −1.34 to 0.37), *p = 0.263*), and BSA (β = 0.77 (95% CI: −1.49 to 3.03), *p = 0.497*) and ATI (β = −1.1 (95% CI: −2.65 to 0.45), *p = 0.159*), were not independently significant. 3D distensibility in the DAo in the native MFS group was significantly correlated with age (β = −0.03 (95% CI: −0.06 to −0.01), *p = 0.014*)*,* BSA (β = −1.38 (95% CI: −2.62 to −0.14), *p = 0.029*) and ATI (β = −1.10 (95% CI: −2.65 to −0.09), *p = 0.034*) in the univariate but not in the multivariate analysis. For RR MFS patients, ATI *(*β = −*1.4 (*95% CI: −*2.39 to −0.41), p= 0.007*) was significantly negatively associated with DAo 3D distensibility and a trend towards lower DAo 3D distensibility was found for patients with a history of valve-sparing root replacement (β = −1.15 (95% CI: −2.37 to 0.06), *p = 0.062*) and Bentall (β = −1.24 (95% CI: −2.48 to 0.00), *p = 0.050*) compared to a history of personalized external aortic root support (PEARS) in the univariate regression analysis.

In HV, male sex (β = −1.03 (95% CI: −1.89 to −0.17), *p = 0.020*) and mean arterial pressure (β = −0.05 (95% CI: −0.09 to −0.01), *p = 0.024)* were identified as independently associated with AAo 3D distensibility in the multivariate regression analysis, while age (β = −0.04 (95% CI: −0.10 to 0.01), *p = 0.112*), BSA (β = 1.34 (95% CI: −1.41 to 4.09), *p = 0.331*), aortic root diameter (β = −0.07 (95% CI: −0.19 to 0.05), *p = 0.250*) and ATI (β = −1.09 (95% CI: −2.82 to 0.64), *p = 0.210*) were not independently significant. Univariate analysis of the DAo in HVs revealed a significant correlation between 3D distensibility and male sex (β = −0.82 (95% CI: −1.42 to −0.22), *p = 0.008*), age (β = −0.06 (95% CI: −0.11 to −0.02), *p = 0.003*), mean arterial pressure (β = −0.04 (95% CI: −0.08 to −0.01), *p = 0.015*), BSA (β = −2.45 (95% CI: −3.97 to −0.94), *p = 0.002*) aortic root diameter (β = −0.10 (95% CI: −0.18 to −0.02), *p = 0.014*) and ATI (β = −1.84 (95% CI: −3.14 to −0.54), *p = 0.007*). These factors were not independently associated in the multivariate analysis.

**Fig. 9:**
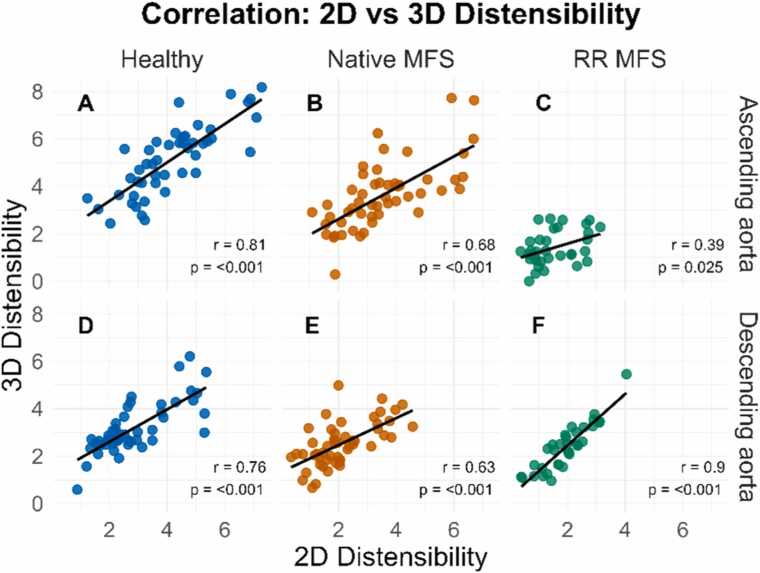
Correlation between 2D and 3D distensibility. Correlation of 2D and 3D distensibility measurements in the ascending (A-C) and descending aorta (D-F) reported separately per study group. *3D* three-dimensional, *2D* two-dimensional, *MFS* Marfan syndrome

2D and 3D distensibility were significantly correlated for all study groups in both the AAo (Healthy: r = 0.81, *p*<0.001; native MFS: r = 0.68, p<0.001; RR MFS: r = 0.39, *p *= 0.025) and DAo (Healthy: r = 0.76, *p*<0.001; native MFS: r = 0.63, *p*<0.001; RR MFS: r = 0.90, *p*<0.001) (Fig. 9).

## 4. Discussion

In this study, we evaluated aortic biomechanics in MFS patients and healthy volunteers using 3D cine bSSFP CMR. Our main findings are: 1) Using a single CMR sequence <5 min, we were able to acquire extensive information on aortic anatomy and dynamic function, allowing for the evaluation of known and novel markers, 2) 3D distensibility is a novel imaging parameter associated with arterial stiffness, and is decreased in MFS compared to HV, 3) Differences in 3D aortic displacement through the cardiac cycle between MFS patients and volunteers are most pronounced at the sinotubular junction level.

2D distensibility is a well-known measure for arterial stiffness [9]. A decrease in 2D distensibility is associated with increasing age and is an independent predictor for all-cause mortality in the general population [20]. In MFS, aortic 2D distensibility is decreased, but an association with increased aortic events could previously not be demonstrated [21], [22], [23], [24]. This may be explained by the fact that 2D methods of calculating distensibility do not account for through-plane motion due to longitudinal displacement [25].

In this work, we propose aortic 3D distensibility as a measurement of arterial stiffness, combining information on 2D distensibility, longitudinal strain, aortic displacement, and diameter, providing one value for these parameters. 3D distensibility, or even volume change, which is the basis of our 3D distensibility measurement, has not been investigated with CMR. Interestingly, we found similar absolute volume change in HV and MFS, while MFS have larger end-diastolic values, indicating less expansion over the cardiac cycle. This is reflected in the 3D distensibility differences between the studied groups.

As aortic root surgery might shift the loading conditions during the cardiac cycle from the ascending aorta to the aortic arch and descending aorta [26], we anticipated that this would result in differences in distensibility parameters of the DAo between native and RR MFS patients. However, we observed that DAo end-diastolic volume (significantly) and absolute volume change (trend) were higher in RR MFS patients. This may indicate that more blood must be buffered in the descending aorta once the AAo is no longer compliant. Nevertheless, if both the volume and the absolute change increase to a similar extent, this would not lead to differences in distensibility.

We found that age was independently associated with a decrease in AAo 3D distensibility in native MFS, but not in RR MFS patients. This is in line with expectations as the stiff aortic graft in the RR MFS group is expected to behave similarly regardless of age, while we know that native tissue gets increasingly stiff with age in the general population and in MFS [27], [28]. Interestingly, we found a trend toward higher DAo 3D distensibility for patients who have a history of PEARS compared to those with a history of valve-sparing root replacement or Bentall procedure. Although this result should be interpreted with caution, as only three patients had a history of PEARS in this study, it might suggest that DAo 3D distensibility is less deteriorated in these patients, compared to those treated with established surgical techniques. This may be explained by the characteristics of the PEARS procedure, in which the native aortic tissue and anatomy are preserved, and the graft is incorporated into the periadventitial tissue [29]. However, studies with more patients with a history of PEARS are warranted to investigate this in more detail.

Furthermore, we found that ATI was negatively associated with 3D distensibility independently of sex, age, BSA, and root diameter in native MFS patients. ATI increases with increasing aortic length, and it is known that with age, the aorta lengthens [27]. A higher ATI has been associated with a more severe MFS phenotype, and AAo length has been associated with a higher incidence of aortic events in an AAo aneurysm [18], [30], [31]. One could postulate that 3D distensibility might also reflect this age-independent relationship with aortic events. We actually anticipated an effect of betablockers or angiotensin-II receptor blocker use on 3D distensibility, but were unable to demonstrate this correlation. However, the reduction of arterial stiffness after betablocker administration is thought to be mainly secondary to blood pressure decrease [32]. Furthermore, studies examining the effects of angiotensin-II receptor blockers on arterial stiffness, independent of blood pressure, have primarily been conducted in hypertensive populations [33]. Blood pressure in our study was generally well-regulated, either with or without medication, and treatment indication was not hypertension. Therefore, it is possible that our dataset configuration was not suitable to demonstrate such a relationship. Additionally, we did not observe a correlation of left ventricular function or aortic regurgitation with 3D distensibility. However, left ventricular function was only mildly decreased in eight patients, and the aortic regurgitation was not more than moderate in a small proportion of the patients. Future studies should evaluate 3D distensibility in patients with markedly reduced left ventricular function and/or aortic regurgitation to determine how these pathologies influence the studied parameters.

We observed a decreased AAo longitudinal strain in both MFS groups (RR MFS: 8.7% and native MFS: 13.4%) compared to HV (15%). A previous study evaluated AAo longitudinal strain longitudinally in 107 native MFS patients and found it to be associated with aortic growth and aortic events, independent of root diameter and known risk factors [24]. They observed a longitudinal strain of 8.7% in MFS patients, which is lower than in our study. However, in their study, the change in centerline length was calculated by registration of three displacements derived from 2D cine bSSFP scans, rather than true 3D imaging, which we used in our study. Bell *et al*. found AAo longitudinal strain derived from 2D oblique coronal bSSFP images to be of 6.7–8.5% from brachiocephalic trunk to sinotubular junction in HV but reported on strain of the aortic cusps separately, which they found to be 22% [34]. It has been hypothesized that longitudinal strain may play a major role in the development of intimal tears in the typical transverse orientation of type A dissections near the sinotubular junction, perpendicular to this main direction of aortic stretch [8], [35], [36]. Indeed, the current exploratory study confirms that most displacement takes place in this proximal region of the aorta, and this is also the region where group differences in displacement were most pronounced. The predictive value of these parameters on aortic dissections remains to be investigated.

Other publications evaluating aortic displacement using 3D cine CMR techniques are scarce [37]. The long acquisition time without acceleration and the absence of 3D cine CMR in clinical protocols likely explain this. One group retrospectively evaluated aortic displacement using dynamic computed tomography angiography (CTA) in non-aneurysmal, thoracic aortic aneurysm, MFS, and RR patients in two studies [22], [25]. Congruent with our results, displacement was significantly decreased for the RR group. However, total displacement was lower in their study, and they were unable to demonstrate a difference between non-aneurysmal volunteers and MFS patients, a difference we did observe in our study. The lower displacement values in these studies may be due to the dynamic CTA's temporal resolution being limited to 10–20 cardiac phases. Fewer cardiac phases risk missing the true peak displacement, leading to underestimation. Furthermore, CMR avoids radiation and contrast, making it more suitable for lifelong MFS follow-up than dynamic CTA.

## 5. Limitations

First, all analyses were performed on the segmentations of the aortic lumen, meaning we do not directly measure deformation and displacement of the aortic wall itself. Further, as this study uses a segmentation-based non-rigid registration method for displacement calculations, its deformation is based on surface shape and uses internal settings, which can be viewed as assumptions about its deformability. It is beneficial that this method is less affected by signal variation in CMR images compared to image-based registration methods and performs well as long as the image quality is sufficient for automated segmentation.

Second, variation in signal intensities of blood in non-contrast 3D cine bSSFP of the aorta as a result of banding artifacts can be an issue for robust scan quality [10]. Indeed, this has resulted in the exclusion of four of the acquired datasets.

Third, peripheral blood pressure used for distensibility calculations in this study is higher compared to the central pulse pressure and it has been suggested that central blood pressure better reflects the hemodynamic stress on target organs [38]. This might have resulted in an underestimation of distensibility.

Also, as follow-up or prognostic data were not acquired, this study can be considered as exploratory.

Lastly, in the current study, cardiac function was not evaluated during the CMR examination. Although we included clinically available echocardiography data, in future studies it would be valuable to evaluate these parameters in more detail during the study visit.

## 6. Conclusion

In this exploratory study, we evaluated 3D distensibility and its separate components 2D distensibility, longitudinal strain and displacement using a single novel CMR sequence in MFS patients in comparison with HV. We observed significant differences between (subgroups of) MFS patients and HV and found correlations with known markers for arterial stiffness. Clinical follow-up data are required to determine if these novel parameters have predictive value for identifying MFS patients at elevated risk of dissection.

## Funding

This study is part of the research program Applied and Engineering Sciences and the project Comprehensive Assessment of 4D Thoracic Aorta Biomechanics Using Novel Cardiac MRI Technology (number 18402), financed by the Dutch Research Council (NWO). EMS acknowledges funding by ITEA Eureka cluster on Software innovation through the SIGNET project number 20052.

## Author contributions

**Daan Bosshardt:** Writing – original draft, Investigation, Formal analysis. **Renske Merton:** Writing – review & editing, Writing – original draft, Methodology, Investigation, Formal analysis. **Bibi A. Schreurs:** Writing – review & editing, Investigation. **Roland R.J. van Kimmenade:** Writing – review & editing. **Aart J. Nederveen:** Writing – review & editing, Conceptualization. **Moniek G.P.J. Cox:** Writing – review & editing. **Arthur J.H.A. Scholte:** Writing – review & editing. **Eric M. Schrauben:** Writing – review & editing, Supervision, Methodology, Investigation, Formal analysis, Conceptualization. **Gustav J. Strijkers:** Writing – review & editing, Supervision, Methodology. **Vivian de Waard:** Writing – review & editing. **Daniëlle Robbers-Visser:** Writing – review & editing, Supervision. **Maarten Groenink:** Writing – review & editing, Supervision, Conceptualization. **Pim van Ooij:** Writing – review & editing, Supervision, Methodology, Formal analysis, Conceptualization.

## Ethics approval and consent

The study is conducted in accordance with GCP, Medical Research Involving Human Subjects Act (WMO), and the Declaration of Helsinki (7th revision, October 2013). The study protocol has been approved by the local ethics committee (Medisch Ethische Toetsingscommissie Amsterdam UMC).

## Declaration of Generative AI and AI-assisted technologies in the writing process

During the preparation of this work, the author used Grammarly and ChatGPT to correct English grammar/spelling and improve readability. After using this tool/service, the author reviewed and edited the content as needed and takes full responsibility for the content of the publication.

## Declaration of competing interests

The author is an Editorial Board Member/Editor-in-Chief/Associate Editor/Guest Editor for this journal and was not involved in the editorial review or the decision to publish this article.

## Data Availability

For this study, we used the Amsterdam UMC ‘PROspective Undersampling in multiple Dimensions’ (PROUD) software patch (https://mriresearch.amsterdam/software/aumcproudpatch/). All data and software are available on reasonable request.

## References

[1] Judge D.P., Dietz H.C. (2005). Marfan's syndrome. Lancet.

[2] Groth K.A., Stochholm K., Hove H., Andersen N.H., Gravholt C.H. (2018). Causes of mortality in the Marfan syndrome (from a Nationwide Register Study). Am J Cardiol.

[3] Mazzolai L., Teixido-Tura G., Lanzi S., Boc V., Bossone E., Brodmann M. (2024). 2024 ESC Guidelines for the management of peripheral arterial and aortic diseases. Eur Heart J.

[4] Kim E.K., Choi S.H., Sung K., Kim W.S., Choe Y.H., Oh J.K. (2014). Aortic diameter predicts acute type A aortic dissection in patients with Marfan syndrome but not in patients without Marfan syndrome. J Thorac Cardiovasc Surg.

[5] Narula N., Devereux R.B., Arbustini E., Ma X., Weinsaft J.W., Girardi L. (2023). Risk of Type B Dissection in Marfan Syndrome: The Cornell Aortic Aneurysm Registry. J Am Coll Cardiol.

[6] Weismann C.G., Hlebowicz J., Åkesson A., Liuba P., Hanseus K. (2022). Comprehensive characterization of arterial and cardiac function in Marfan syndrome-can biomarkers help improve outcome?. Front Physiol.

[7] Sakai L.Y., Keene D.R., Renard M., De Backer J. (2016). FBN1: the disease-causing gene for Marfan syndrome and other genetic disorders. Gene.

[8] Hirst A.E., Johns V.J., Kime S.W. (1958). Dissecting aneurysm of the aorta: a review of 505 cases. Medicine.

[9] O'Rourke M.F., Staessen J.A., Vlachopoulos C., Duprez D., Plante G.E. (2002). Clinical applications of arterial stiffness; definitions and reference values. Am J Hypertens.

[10] Merton R., Bosshardt D., Strijkers G.J., Nederveen A.J., Schrauben E.M., van Ooij P. (2024). Reproducibility of 3D thoracic aortic displacement from 3D cine balanced SSFP at 3 T without contrast enhancement. Magn Reson Med.

[11] Merton R., Bosshardt D., Strijkers G.J., Nederveen A.J., Schrauben E.M., van Ooij P. (2024). Assessing aortic motion with automated 3D cine balanced steady state free precession cardiovascular magnetic resonance segmentation. J Cardiovasc Magn Reson.

[12] Loeys B.L., Dietz H.C., Braverman A.C., Callewaert B.L., De Backer J., Devereux R.B. (2010). The revised Ghent nosology for the Marfan syndrome. J Med Genet.

[13] Gottwald L.M., Peper E.S., Zhang Q., Coolen B.F., Strijkers G.J., Nederveen A.J. (2020). Pseudo-spiral sampling and compressed sensing reconstruction provides flexibility of temporal resolution in accelerated aortic 4D flow MRI: a comparison with k-t principal component analysis. NMR Biomed.

[14] Feng L., Grimm R., Block K.T., Chandarana H., Kim S., Xu J. (2014). Golden-angle radial sparse parallel MRI: combination of compressed sensing, parallel imaging, and golden-angle radial sampling for fast and flexible dynamic volumetric MRI. Magn Reson Med.

[15] Isensee F., Jaeger P.F., Kohl S.A.A., Petersen J., Maier-Hein K.H. (2021). nnU-Net: a self-configuring method for deep learning-based biomedical image segmentation. Nat Methods.

[16] Carr J.C., Beatson R.K., Cherrie J.B., Mitchell T.J., Fright W.R., McCallum B.C. (2001). Reconstruction and representation of 3D objects with radial basis functions. Proc 28th Annu Conf Comput Graph Interact Tech Assoc Comput Mach.

[17] Audenaert E.A., Van Houcke J., Almeida D.F., Paelinck L., Peiffer M., Steenackers G. (2019). Cascaded statistical shape model based segmentation of the full lower limb in CT. Comput Methods Biomech Biomed Engin.

[18] Franken R., El Morabit A., de Waard V., Timmermans J., Scholte A.J., van den Berg M.P. (2015). Increased aortic tortuosity indicates a more severe aortic phenotype in adults with Marfan syndrome. Int J Cardiol.

[19] James G., Witten D., Hastie T., Tibshirani R. (2013).

[20] Redheuil A., Wu C.O., Kachenoura N., Ohyama Y., Yan R.T., Bertoni A.G. (2014). Proximal aortic distensibility is an independent predictor of all-cause mortality and incident CV events: the MESA study. J Am Coll Cardiol.

[21] van Andel M.M., de Waard V., Timmermans J., Scholte A., van den Berg M.P., Zwinderman A.H. (2021). Aortic distensibility in Marfan syndrome: a potential predictor of aortic events?. Open Heart.

[22] Kim T., Tjahjadi N.S., He X., van Herwaarden J.A., Patel H.J., Burris N.S. (2023). Three-dimensional characterization of aortic root motion by vascular deformation mapping. J Clin Med.

[23] Nollen G.J., Groenink M., Tijssen J.G., Van Der Wall E.E., Mulder B.J. (2004). Aortic stiffness and diameter predict progressive aortic dilatation in patients with Marfan syndrome. Eur Heart J.

[24] Guala A., Teixido-Tura G., Rodriguez-Palomares J., Ruiz-Munoz A., Dux-Santoy L., Villalva N. (2019). Proximal aorta longitudinal strain predicts aortic root dilation rate and aortic events in Marfan syndrome. Eur Heart J.

[25] Tjahjadi N.S., Kim T., Marway P.S., Jorge C.A.C., Baker T.J., Hazenberg C. (2025). Three-dimensional assessment of ascending aortic stiffness, motion, and growth in ascending thoracic aortic aneurysm. Eur Heart J Imaging Methods Pract.

[26] Thomas R., Dhanekula A.S., Byers P., Flodin R., DeRoo S., Shalhub S. (2024). Elective root replacement increases the risk of type B dissection in patients with Marfan syndrome. J Thorac Cardiovasc Surg.

[27] Morrison T.M., Choi G., Zarins C.K., Taylor C.A. (2009). Circumferential and longitudinal cyclic strain of the human thoracic aorta: age-related changes. J Vasc Surg.

[28] Prakash A., Adlakha H., Rabideau N., Hass C.J., Morris S.A., Geva T. (2015). Segmental aortic stiffness in children and young adults with connective tissue disorders: relationships with age, aortic size, rate of dilation, and surgical root replacement. Circulation.

[29] Verbrugghe P., Verbeken E., Pepper J., Treasure T., Meyns B., Meuris B. (2013). External aortic root support: a histological and mechanical study in sheep. Interact Cardiovasc Thorac Surg.

[30] Wu J., Zafar M.A., Li Y., Saeyeldin A., Huang Y., Zhao R. (2019). Ascending aortic length and risk of aortic adverse events: the neglected dimension. J Am Coll Cardiol.

[31] Heuts S., Adriaans B.P., Rylski B., Mihl C., Bekkers S., Olsthoorn J.R. (2020). Evaluating the diagnostic accuracy of maximal aortic diameter, length and volume for prediction of aortic dissection. Heart.

[32] Mahmud A., Feely J. (2008). Beta-blockers reduce aortic stiffness in hypertension but nebivolol, not atenolol, reduces wave reflection. Am J Hypertens.

[33] Peng F., Pan H., Wang B., Lin J., Niu W. (2015). The impact of angiotensin receptor blockers on arterial stiffness: a meta-analysis. Hypertens Res.

[34] Bell V., Mitchell W.A., Sigurðsson S., Westenberg J.J., Gotal J.D., Torjesen A.A. (2014). Longitudinal and circumferential strain of the proximal aorta. J Am Heart Assoc.

[35] Kefalidi E., Angouras D.C., Sokolis D.P. (2022). Regional and directional variations in the layer-specific resistance to tear propagation in ascending thoracic aortic aneurysms. J Biomech.

[36] Beller C.J., Labrosse M.R., Thubrikar M.J., Robicsek F. (2008). Finite element modeling of the thoracic aorta: including aortic root motion to evaluate the risk of aortic dissection. J Med Eng Technol.

[37] Lantz J., Dyverfeldt P., Ebbers T. (2014). Improving blood flow simulations by incorporating measured subject-specific wall motion. Cardiovasc Eng Technol.

[38] Kollias A., Lagou S., Zeniodi M.E., Boubouchairopoulou N., Stergiou G.S. (2016). Association of central versus brachial blood pressure with target-organ damage: systematic review and meta-analysis. Hypertension.

