# Supplementary material for: Three-dimensional distensibility of the aorta derived from four-dimensional cardiovascular magnetic resonance in young and middle-aged adults with Marfan syndrome
Source: J Cardiovasc Magn Reson. 2025 Oct 29;27(2):101975. doi: 10.1016/j.jocmr.2025.101975 (PMC12673023; doi:10.1016/j.jocmr.2025.101975)
Supplement: Supplementary file 1 — Supplementary material [file mmc1.docx]

**Suplementary material figure/GIF**

**
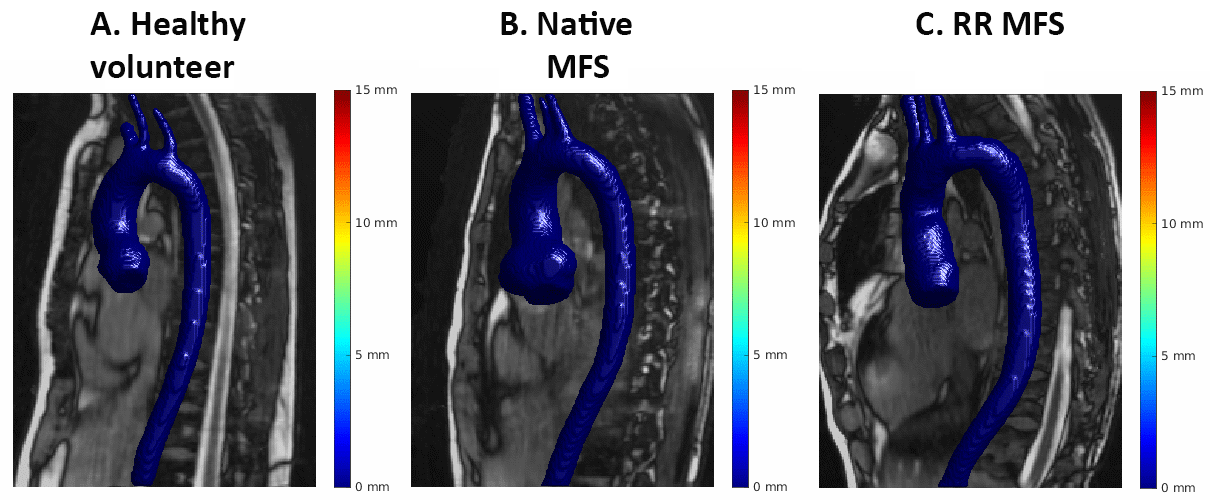
**

**Supplementary material S2**

**Table 1.** Linear regression for Marfan syndrome patients without a history of aortic root surgery and aortic 3D distensibility

| Native MFS patients | | | | | | | | |
| --- | --- | --- | --- | --- | --- | --- | --- | --- |
| Ascending aorta 3D distensibility | | | | | **Descending aorta 3D distensibility** | | | |
|  | **Univariate** | | **Multivariate** | | **Univariate** | | **Multivariate** | |
| Name variable | **β (95% CI)** | **P-value** | **β (95% CI)** | **P-value** | **β (95% CI)** | **P-value** | **β (95% CI)** | **P-value** |
| Male sex | -1.08 (-1.83 – -0.33) | *0.006* | -0.48 (-1.34 – 0.37) | *0.263* | -0.43 (-0.94 – 0.08) | *0.096* |  |  |
| Age inclusion | -0.07 (-0.11 – -0.04) | *0.000* | -0.06 (-0.10 – -0.01) | *0.014* | -0.03 (-0.06 – -0.01) | *0.014* | -0.02 (-0.05 – 0.01) | *0.243* |
| Betablocker use | -0.21 (-1.05 – 0.63) | *0.620* |  |  | 0.16 (-0.38 – 0.70) | *0.545* |  |  |
| ARB use | -0.76 (-1.55 – 0.03) | *0.058* |  |  | -0.47 (-0.98 – 0.05) | *0.073* |  |  |
| FBN1 dominant negative  Dominant negative | 0.4 (-0.58 – 1.39) | *0.414* |  |  | -0.18 (-0.81 – 0.46) | *0.577* |  |  |
| FBN1 effect unknown | 0.97 (-0.23 – 2.16) | *0.110* |  |  | 0.4 (-0.36 – 1.17) | *0.296* |  |  |
| Mildly decreased LVF | 1.42 (-0.63 – 3.47) | *0.170* |  |  | 1.06 (-0.25 – 2.37) | *0.112* |  |  |
| Mild AR | 0.62 (-1.11 – 2.36) | *0.473* |  |  | -0.12 (-1.23 – 0.98) | *0.824* |  |  |
| Moderate AR | 0.39 (-1.72 – 2.49) | *0.713* |  |  | 0.91 (-0.43 – 2.25) | *0.177* |  |  |
| MAP | -0.02 (-0.07 – 0.03) | *0.447* |  |  | -0.03 (-0.06 – 0.01) | *0.113* |  |  |
| BSA | -2.25 (-4.16 – -0.34) | *0.022* | 0.77 (-1.49 – 3.03) | *0.497* | -1.38 (-2.62 – -0.14) | *0.029* | -0.96 (-2.24 – 0.33) | *0.140* |
| Heart rate | -0.01 (-0.04 – 0.02) | *0.546* |  |  | -0.02 (-0.04 – 0.00) | *0.089* |  |  |
| Aortic root diameter | -0.11 (-0.19 – -0.03) | *0.008* | -0.1 (-0.19 – -0.02) | *0.018* | -0.04 (-0.10 – 0.01) | *0.131* |  |  |
| ATI | -1.73 (-3.30 – -0.16) | *0.031* | -1.1 (-2.65 – 0.45) | *0.159* | -1.1 (-2.11 – -0.09) | *0.034* | -0.67 (-1.77 – 0.43) | *0.227* |

Variables with a p-value <0.05 in the univariate regression were entered into the multivariate regression analysis.

AR: aortic valve regurgitation, ARB: angiotensin-II receptor blocker, ATI: aortic tortuosity index, β: coefficient, BSA: body surface area, CI: confidence interval, FBN1: Fibrillin-1 protein, function, LVF: left ventricular function, MAP: Mean arterial pressure, Native MFS: Marfan syndrome patient without a history of aortic root surgery.

**Table 2.** Linear regression for Marfan syndrome patients with a history of aortic root surgery and aortic 3D distensibility

| RR MFS patients | | | | | | | | |
| --- | --- | --- | --- | --- | --- | --- | --- | --- |
| Ascending aorta 3D distensibility | | | | | **Descending aorta 3D distensibility** | | | |
|  | **Univariate** | | **Multivariate** | | **Univariate** | | **Multivariate** | |
| Name variable | **β (95% CI)** | **P-value** | **β (95% CI) CI)** | **P-value** | **β (95% CI)** | **P-value** | **β (95% CI)** | **P-value** |
| Male sex | -0.19 (-0.75 – 0.36) | *0.488* |  |  | -0.33 (-1.08 – 0.41) | *0.369* |  |  |
| Age inclusion | 0.02 (-0.02 – 0.06) | *0.346* |  |  | -0.04 (-0.09 – 0.01) | *0.102* |  |  |
| Betablocker use | -0.06 (-0.60 – 0.48) | *0.828* |  |  | -0.02 (-0.74 – 0.71) | *0.963* |  |  |
| ARB use | -0.35 (-0.87 – 0.17) | *0.179* |  |  | -0.43 (-1.13 – 0.28) | *0.225* |  |  |
| Mildly decreased LVF | 0.47 (-0.19 – 1.13) | *0.158* |  |  | -0.31 (-1.22 – 0.61) | *0.498* |  |  |
| Mild AR | 0.06 (-0.56 – 0.67) | *0.848* |  |  | 0.36 (-0.46 – 1.18) | *0.374* |  |  |
| FBN1 variant type |  |  |  |  |  |  |  |  |
| Haploinsufficient (ref) |  |  |  |  |  |  |  |  |
| Dominant negative  Dominant negative | 0.03 (-0.54 – 0.60) | *0.909* |  |  | -0.29 (-1.05 – 0.47) | *0.441* |  |  |
| Effect unknown | 0.32 (-0.53 – 1.18) | *0.446* |  |  | -0.51 (-1.66 – 0.64) | *0.372* |  |  |
| MAP | 0.00 (-0.04 – 0.03) | *0.963* |  |  | 0.00 (-0.05 – 0.05) | *0.967* |  |  |
| BSA | -0.79 (-1.88 – 0.30) | *0.151* |  |  | -1.22 (-2.68 – 0.23) | *0.096* |  |  |
| Heart rate | 0.01 (-0.01 – 0.04) | *0.306* |  |  | -0.01 (-0.05 – 0.02) | *0.461* |  |  |
| ATI | -0.45 (-1.27 – 0.36) | *0.267* |  |  | -1.4 (-2.39 – -0.41) | *0.007* |  |  |
| Years since RR | 0.02 (-0.03 – 0.06)  0.404 | *0.404* |  |  | -0.04 (-0.1 – 0.02) | *0.143* |  |  |
| RR Procedure |  |  |  |  |  |  |  |  |
| PEARS (reference) | - | *-* |  |  | - | *-* |  |  |
| Bentall | 0.57 (-0.39 - 1.52) | *0.233* |  |  | -1.24 (-2.48 – 0.00) | *0.050* |  |  |
| VSRR | 0.24 ( -0.69 – 1.17) | *0.600* |  |  | -1.15 (-2.37 – 0.06) | *0.062* |  |  |

Variables with a p-value <0.05 in the univariate regression were entered into the multivariate regression analysis.
AR: aortic valve regurgitation, ARB: angiotensin-II receptor blocker, ATI: aortic tortuosity index, β: coefficient, BP: Blood pressure, BSA: body surface area, CI: confidence interval, FBN1: Fibrillin-1 protein, LVF: left ventricular function, MAP: Mean arterial pressure, PEARS: Personalized External Aortic Root Support, RR MFS: Marfan syndrome patient with a history of aortic root surgery, VSRR: Valve Sparing Aortic Root Replacement

**Table 3.** Linear regression for healthy volunteers without aortic surgery and aortic 3D distensibility

| Healthy volunteers | | | | | | | | |
| --- | --- | --- | --- | --- | --- | --- | --- | --- |
| Ascending aorta 3D distensibility | | | | | **Descending aorta 3D distensibility** | | | |
|  | **Univariate** | | **Multivariate** | | **Univariate** | | **Multivariate** | |
| Name variable | **β (95% CI)** | **P-value** | **β (95% CI)** | **P-value** | **β (95% CI)** | **P-value** | **β (95% CI)** | **P-value** |
| Male sex | -1.48 (-2.22 – -0.73) | *<0.001* | -1.03 (-1.89 – -0.17) | *0.020* | -0.82 (-1.42 – -0.22) | *0.008* | -0.36 (-1.14 – 0.43) | *0.363* |
| Age inclusion | -0.10 (-0.15 – -0.05) | *0.001* | -0.04 (-0.10 – 0.01) | *0.112* | -0.06 (-0.11 – -0.02) | *0.003* | -0.04 (-0.09 – 0.01) | *0.086* |
| MAP | -0.09 (-0.13 – -0.05) | *<0.001* | -0.05 (-0.09 – -0.01) | *0.024* | -0.04 (-0.08 – -0.01) | *0.015* | -0.01 (-0.05 – 0.03) | *0.670* |
| BSA | -3.61 (-5.59 – -1.62) | *0.001* | 1.34 (-1.41 – 4.09) | *0.331* | -2.45 (-3.97 – -0.94) | *0.002* | -0.96 (-3.46 – 1.54) | *0.443* |
| Heart rate | -0.01 (-0.06 – 0.03) | *0.549* |  |  | 0.01 (-0.03 – 0.04) | *0.650* |  |  |
| Aortic root diameter | -0.20 (-0.29 – -0.10) | *<0.001* | -0.07 (-0.19 – 0.05) | *0.250* | -0.10 (-0.18 – -0.02) | *0.014* | 0.02 (-0.09 – 0.13) | *0.723* |
| ATI | -3.12 (-4.77 – -1.47) | *<0.001* | -1.09 (-2.82 – 0.64) | *0.210* | -1.84 (-3.14 – -0.54) | *0.007* | -0.82 (-2.40 – 0.76) | *0.301* |

Variables with a p-value <0.05 in the univariate regression were entered into the multivariate regression analysis.
ATI: aortic tortuosity index, β: coefficient, BSA: body surface area, CI: confidence interval MAP: Mean arterial pressure.
